# Injectable self-healing hydrogel fabricated from antibacterial carbon dots and ɛ-polylysine for promoting bacteria-infected wound healing

**DOI:** 10.1186/s12951-022-01572-w

**Published:** 2022-08-11

**Authors:** Chengjian Mou, Xinyuan Wang, Jiahui Teng, Zhigang Xie, Min Zheng

**Affiliations:** 1 School of Chemistry and Life Science, Advanced Institute of Materials Science, Changchun University of Technology, 2055 Yanan Street, Changchun, 130012 Jilin People’s Republic of China; 2 State Key Laboratory of Polymer Physics and Chemistry, Changchun Institute of Applied Chemistry, Chinese Academy of Sciences, 5625 Renmin Street, Changchun, 130022 Jilin People’s Republic of China

**Keywords:** Hydrogel, Negatively charged carbon dots, Antibacterial, Wound healing, ɛ-polylysine

## Abstract

Developing highly efficient pharmaceuticals to eradicate pathogens and facilitate wound healing is of great concern. Despite some cationic carbon dots (CDs) have been used for sterilization, hardly any anionic CDs with antimicrobial activity have appeared. In the present work, we engineered a string of anionic CDs (especially CD31) as valid broad-spectrum bactericides to kill bacteria. Furthermore, CD31 conjugated with ɛ-polylysine (Plys) to construct injectable, and self-healing hydrogel (CD-Plys) that possess the advantages of remarkable broad spectrum antibacterial activity, excellent wound healing ability and satisfied biocompatibility. CD-Plys could dramatically accelerate wound healing with epithelization and enhanced angiogenesis. Taken together, this work provides a two-pronged strategy to explore CDs-based antimicrobial agents for disease therapy and tissue engineering.

**Supplementary Information:**

The online version contains supplementary material available at 10.1186/s12951-022-01572-w.

## Introduction

Bacterial infection is a major risk to worldwide people’s lives, therefore, exploiting innovative strategies to combat microorganisms is highly needed. Although some antibiotics have successfully inhibited bacterial diseases, their abuse aggravates antibiotic resistance of microbes [1–3]. The emergence of nanotechnology provides an alternative strategy to design non-antibiotic bactericides. Multitudinous nanoscale germicides, such as noble metal (eg. Au, Ag, Pd, Ru, Pt) [4–12] nanoparticles (NPs), metallic oxide [13–17] (eg. ZnO, TiO_2_ and CuO) NPs, and carbonaceous nanomaterials [18–20] have been exploited as antimicrobial alternatives, which exhibit high potency with broad spectrum antibacterial activity. However, the clinical applications of these nanofungicides may be hindered due to their inherent cytotoxicity, high cost or long-term retention. Therefore, it is crucial to develop nontoxic and environmentally benign antimicrobial agents for effectively killing bacteria, either from the academic research or clinic therapy point of view.

Carbon dots (CDs) have wide applications in bioimaging [21–31], sensing [32–37], drug/dye/protein delivery [38–41] and cancer therapy [42–50], owning to their excellent characteristics including small size, easy surface functionalization, high stability, strong hydrophilicity, good biocompatibility and low toxicity. By contrast, the potential antibacterial capacity of CDs has been less explored [51–53], although CDs possess the advantages of durable and environmentally friendly than metal-containing nanofungicides and traditional antibiotics. Furthermore, most of CDs have to utilize external reagents or equipment to achieve bacteriostasis. For instance, Sun et al. has reported CDs can effectively kill bacteria under the exposure to visible light [54]. Huang et al. prepared halogen/nitrogen-doped CDs to kill bacteria by producing reactive oxygen species upon LED irradiation [55]. Zhang et al. prepared cerium-doped CDs for wound healing under UV excitation [56]. Qu et al. enhanced the antibacterial performance of CDs at the aid of H_2_O_2_ [57]. However, adding agents or light illumination is relatively sophisticated and might bring about accidental injury to certain tissues. Therefore, exploiting CDs-based nanofungicides to kill bacteria and heal wound infections with no external activation is becoming the focus of concern. It is generally believed that highly positively charged CDs can easily interact with negatively charged bacteria and directly disrupt the bacterial cell membranes via electrostatic interaction [58]. For example, quaternized CDs constructed by Wu’s group inactivated Gram-positive bacteria [51, 59]. Quaternized CDs fabricated by Zhao et. al could kill both Gram-positive and Gram-negative bacteria [60]. Supercationic CDs synthesized by Jian et al. could effectively eliminate non-multidrug-resistant and multidrug-resistant bacteria, and methicillin-resistant S. aureus [61]. Nevertheless, as far as we know, rare negatively charged CDs with remarkable antibacterial activity have been reported.

Hydrogels are three-dimensional soft materials with porous structures, excellent water absorption ability, and good biocompatibility. The practical applications of traditional hydrogels are usually impeded by the weaknesses of poor mechanical properties and limited functions. Lately, nanohydrogels those are constructed from nanomaterials including metal-containing NPs [62–67], metal-free NPs [68, 69] and metal organic frameworks [70, 71], integrate the functions of nanoagents and macromolecules, significantly enrich the potential applications of hydrogels. What’s more, CDs-based nanohydrogels integrate the characteristics inherited from CDs and polymers, have shown wide applications in sensing [72–74], environmental pollutants removal [75], microbial elimination [76], cartilage regeneration [77], and supercapacitors [78] to name just a few. Unfortunately, few reports on CDs antibacterial hydrogels were mentioned so far. Therefore, there is an urgent need to develop a facile mode to construct antibacterial CDs-based hydrogel for killing bacteria and healing tissue infections effectively.

**Scheme 1:**
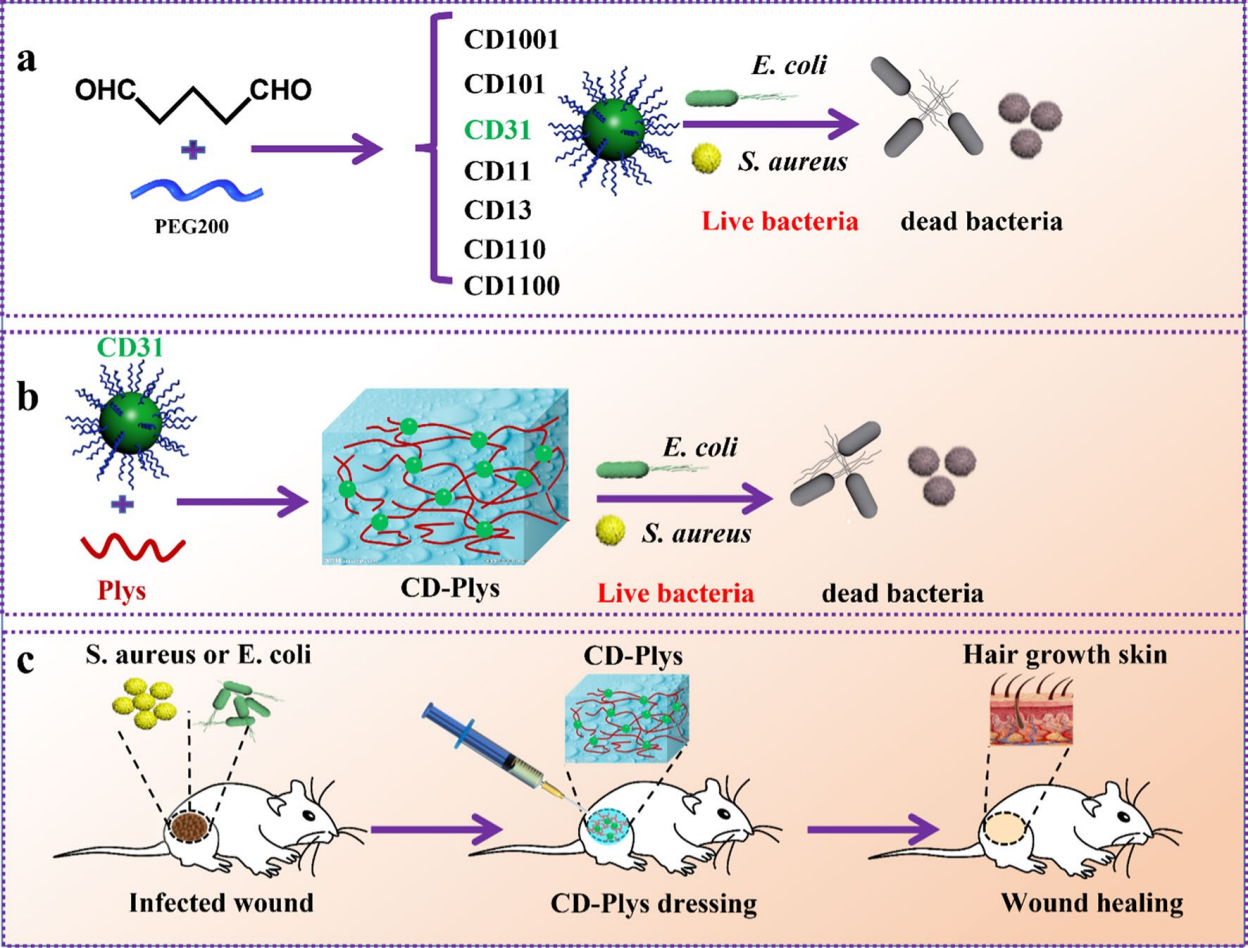
Schematic illustration for (**a**) The preparation of CDs and their antimicrobial ctivity against Gram-negative and Gram-positive bacteria. **b** Synthesis of CD-Plys hydrogel and its inhibitory effect on *E. coli* and *S. aureus*. **c** CD-Plys hydrogel acts as a wound dressing to prevent bacterial infection and promote wound healing on mouse models

Herein, Glutaraldehyde (GA), a widely used cross-linking agent and disinfectant, acted as a precursor to react with PEG_200_ at the volume ratio of 100:1, 10:1, 3:1, 1:1, 1:3, 1:10, 1:100, and obtained a series of negatively charged CDs denoted as CD1001, CD101, CD31, CD11, CD13, CD110 and CD1100 (Scheme 1a). The zeta potential of CD1001, CD101, CD31, CD11, CD13, CD110 and CD1100 was -13.2, -9.8, -9.0, -11.1, -14.9, -20.4 and -20.4 mV, respectively (Additional file 1: Fig. S1). Among the CDs, CD31 exhibited the most significant inhibitory effect on *E. coli* and *S. aureus*, with the minimum inhibitory concentration (MIC) of 64 and 32 μg/mL, respectively. The aldehyde groups on the surface of CDs could destroy the bacterial membrane and efficaciously eradicate pathogens [79, 80]. Meanwhile, the other fungicide, ɛ-Polylysine (Plys) also could eliminate *E. coli* and *S. aureus* with MIC of 128 µg/mL. The win–win co-operation of these two germicides was achieved by the conjugation of CD31 with Plys to form stable, injectable, self-healing and antibacterial CD-Plys hydrogel (Scheme 1b). CD-Plys exhibited enhanced broad-spectrum antimicrobial efficacy (Scheme 1b–c) and can automatically adapt to different conditions of wounds to promote wound closure, avoid infection and contaminating, supply wound healing with three-dimensional physiological environment and facilitate wound healing (Scheme 1c). The hemolysis and cytotoxicity assays verified that CD-Plys has good biocompatibility. Moreover, in vivo wound healing experiments demonstrated that CD-Plys could completely cover the whole wounds and promote full-thickness skin wound healing. Therefore, CD-Plys has great potential for application in bacteria-induced wound infection and tissue reconstruction.

## Materials and methods

### Synthesis of CDs

Taking CD31 as an example, GA (300 µL) and PEG_200_ (100 µL) were dissolved in 5 mL of ethanol. The solution was transferred to a Teflon-lined autoclave and heated for 140 min at 150 °C. After cooled to room temperature, the resultant brown solid was dispersed in deionized water and centrifuged at 10,000 rpm for 20 min to remove carbon aggregates The crude product was dialyzed against water by a dialysis bag (cutoff Mn: 3.5 kDa) for 24 h, and the brown CDs were obtained by freeze-drying.

The preparation and purification process of CD1001, CD101, CD11, CD13, CD110 and CD1100 was similar with that of CD31, except the volume ratio of GA and PEG_200_ was 100:1 (1000 µL:10 µL), 10:1 (100 µL:10 µL), 3:1 (300 µL:100 µL), 1:1 (100 µL:100 µL), 1:3 (100 µL:300 µL), 1:10 (10 µL:100 µL), 1:100 (10 µL:1000 µL), respectively.

### Synthesis of CD-Plys hydrogel.

The aqueous solution of Plys (400 mg/mL, 1 mL) and CDs (200 mg/mL, 1 mL) was added to a vial and the mixture was kept at room temperature for 40 min for gelation, and the gel viscosity was verified by the method from “stable to inversion”.

### Materials

ε-Polylysine (Plys) was purchased from Meilun Biotechnology Co., Ltd. Agar, peptone, and yeast powder were purchased from CSI Biochemical Technology Co., Ltd. Sodium chloride, sodium hydroxide, glutaraldehyde, absolute ethanol, and polyethylene glycol (PEG_200_) were purchased from Sinopharm Chemical Reagent Co., Ltd. The live/dead cell double staining kit was purchased from Jiangsu KGI Biotechnology Co., Ltd. The MTT Cell Proliferation and Cytotoxicity Assay Kit was purchased from Shanghai Yuanye Biological Technology Co., Ltd. The cell culture medium (DMEM) was purchased from Gibco. SYTO 9/PI kit was purchased from Jiangsu KeyGEN Biotechnology Co., Ltd. LB liquid medium (peptone 1%, yeast powder 0.5%, sodium chloride 1%). LB solid medium (peptone 1%, yeast powder 0.5%, sodium chloride 1%, agar powder 1.5%).

### Characterization

Fourier transform infrared (FTIR) spectroscopy was obtained by a Bruker Vertex 70 IR spectrophotometer. UV–Vis absorption spectra were performed on a UV-2450PC spectrophotometer (Shimadzu, Japan). The fluorescence spectra were measured by LS-55 fluorescence spectrometer (Perkin-Elmer, USA). Transmission electron microscopy (TEM) images were acquired by JEM-1011 electron microscope (JEOL Co., Japan). Morphology characterization of CD-Plys was performed on scanning electron microscopy (SEM, Micromeritics FEI PHILIPS) with an accelerating voltage of 10 kV. Samples were mounted onto the specimen stubs by means of a conductive double-sided adhesive tape and sputtered with gold for 40 s. X-ray diffraction (XRD) patterns of CD31, Plys and CD-Plys were performed on Bruker D8 diffractometer. The zeta potentials of CD31, Plys and CD-Plys were measured by Zeta-sizer Nano ZS (Malvern Instruments Ltd., UK). The rheometer (Anton Paar, Physical MCR 302) was carried out to evaluate dynamic rheology behavior of CD-Plys. The bacterial confocal images of CD31, Plys and CD-Plys were obtained using Zeiss confocal laser microscope (ZEISS LSM 700).

### In vitro antibacterial activities of CDs and Plys

The suspended *E. coli* and *S. aureus* cells post-treatment with various concentration of the CDs or Plys were incubated at 37 °C for 30 min, respectively. The 200 µL solution of samples co-incubated with bacteria were surface-plated on Luria–Bertani agar plates, and the plates were incubated at 37 °C for 24 h. The number of colonies was counted, and the viable cell numbers of the treated samples and the controls were calculated in CFU/mL.

### Rheological properties of CD-Plys hydrogel

We measured the storage modulus (G') and loss modulus (G'') of CD-Plys hydrogel by a rheometer. The hydrogel sample was placed in the center of a 25 mm cone, the upper plate was placed at 0.5 mm, and the temperature was set at 37 ℃. First, the angular frequency was fixed, and the G' of the sample within the range of the shear stress of 0.01 ~ 100% was measured. We took the shear stress as the abscissa and G' as the ordinate to determine the linear viscoelasticity. Then we selected the angular frequency from 0.1 to 100 rad/s, measured the G’ and G” of the sample, and used the angular frequency as the abscissa and G’ and G” as the ordinate to plot the rheology curve.

### Hemolysis assay

Blood was incubated with CD-Plys hydrogel, PBS and Triton X-100 at 37 °C for 60 min, respectively. After centrifugation, the absorbance of the supernatant in each group at 540 nm was determined.

The hemolysis (%) was calculated by the following equation:

$$\begin{gathered} {\text{Hemolysis }}\left( \% \right) \, = \, [({\text{OD}}_{{\text{x}}} - {\text{OD}}_{{\text{o}}} )/({\text{OD}}_{{\text{y}}} - {\text{OD}}_{{\text{o}}} )] \times {1}00 \hfill \\ \hfill \\ \hfill \\ \end{gathered}$$

where OD_x_, OD_o_ and OD_y_ are the absorbance values of the hydrogel, diluted blood in PBS and diluted blood in Triton X-100, respectively.

### MTT assay

The cytotoxicities of CDs and Plys were evaluated toward L929 fibroblast cell lines by MTT assay. Firstly, 1.0 × 10^4^ cells were seeded into a 96 well plate in DMEM and in a humidified 5% CO_2_ incubator at 37 °C. Afterwards L929 fibroblast cells were treated for 24 h with different concentrations of CD31 or Plys DMEM solution. Next, 10 μL of MTT solution was added to each well and incubated for 4 h in the dark. Dimethyl sulfoxide was added to dissolve the MTT formazan crystals. Finally, the absorbance was measured at 490 nm using a microplate reader (Bio TeKtronixELX808TM USA). All the assays were conducted in four parallel groups.

The cytotoxicity of CD-Plys was evaluated by direct contact with L929 cells. 100 µL of CD-Plys solution was introduced to each well of 96-well cell culture plates. After 60 min, CD-Plys hydrogel formed and washed with sterile PBS solution. Thereafter, L929 cells were seeded on CD-Plys at a density of 1.0 × 10^4^ cells per well. The cells were incubated for 12, 24 and 48 h in a humidified 5% CO_2_ incubator at 37 °C, and the cell viability was determined by MTT assay. OD values at 490 nm were measured by a microplate reader. All the experiments were performed in four parallel groups.

### In vitro antibacterial assays

The antibacterial activity of CD-Plys hydrogel was evaluated using an inhibition zone assay against *E. coli* and *S. aureus*. First, the density of *E. coli* or *S. aureus* was adjusted to 10^5^ CFU/mL and then the bacteria were spread on the agar surface, respectively. Afterward, the hydrogel were placed on the center of agar plates, and co-cultured with *E. coli* or *S. aureus* for 24 h at 37 °C. The antibacterial effect was compared by the diameter of the inhibition zone.

### Bacteria live/dead staining assay

The bacteria and CD-Plys were cocultured for 4 h, and stained with SYTO 9 (green fluorescence) and PI (red fluorescence) for 30 min in the dark. Then the mixture was observed by CarlZeiss LSM 710 confocal laser scanning microscope (Zurich, Switzerland).

### In vivo infected wound healing

All animal procedures were conducted under the guidelines approved by the Animal Ethics Committee of Changchun Institute of Applied Chemistry, Chinese Academy of Sciences (No. 2021–0004). The infected wound on the skin of the mice were created to evaluate the in vivo antibacterial and healing abilities of CDs, Plys, CD-Plys. In total, the 24 female mice were randomly divided into eight groups. After the fur of the dorsal skin in all mice was shaved, full-thickness skin excisional round wounds (8 mm in diameter) were created on the dorsal of mice. The wound was infected with *E. coli* (1 × 10^5^ CFU in 20 L PBS) or *S. aureus* (1 × 10^5^ CFU in 20 L PBS), then treated with PBS (control group), CDs, Plys and CD-Plys, respectively. The wound was covered with sterile gauze, and all the above operations were performed under pentobarbital anesthesia. In addition, the camera was used to record the pictures of wound, and the data were analyzed with image J software at 0, 3, 5, and 7 days after treatment. The closure rate of wound in the four groups at each time point was calculated by the formula:

$${\text{healing rate }}\left( \% \right) \, = [({\text{A}}_{0} - {\text{A}}_{{\text{t}}} )/{\text{A}}_{0} ] \times {1}00\%$$

A_0_ represents the initial wound area and A_t_ represents the residual wound area at each time point.

### Skin colony counting method

One days after treatment, the wound tissues were harvested and soaked in sterile saline (1 mL) to obtain the bacteria containing solutions. Aliquots of diluted samples were placed on agar for the growth of bacteria and the colonies were counted for analysis after the solutions were cultured at 37 °C.

### Histological analysis

After 7 days of treatment, all the mice were sacrificed, and the tissues of the wound skins were harvested. The photographs of hematoxylin and eosin (H&E) stained sections were taken by using an optical microscope (Nikon, Japan).

## Results and discussion

### Synthesis and in vitro antibacterial activities of CDs

**Fig. 1:**
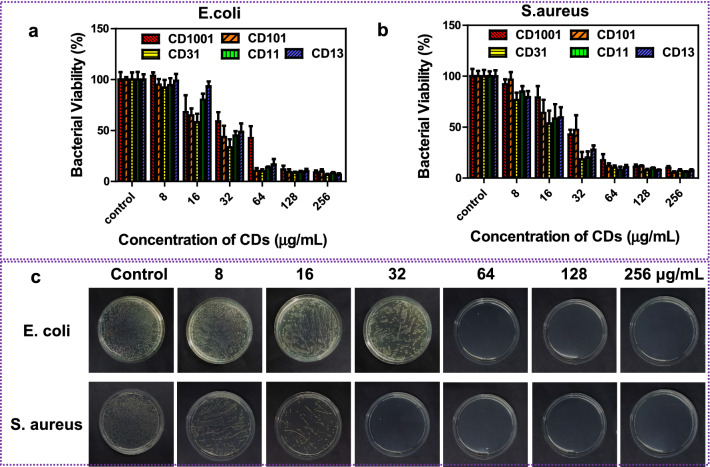
In vitro antibacterial assay of CDs. Antimicrobial assay of CDs against **a** *E. coli* and **b** *S. aureus*. Data expressed as mean ± SD (*n* = 3), *P < 0.05; **P < 0.01; ***P < 0.001. P values represent the outcome of Multiple Comparisons in Analysis of Variance (ANOVA). **c** LB agar plates of *E. coli* or *S. aureus* inoculated with CD31 for 2 h

CDs were synthesized from various volume ratio of GA and PEG_200_ via solvothermal method at 150 °C for 140 min (see supporting information). As a proof of concept, we selected *E. coli* and *S. aureus* as models for Gram-negative and Gram-positive bacteria to estimate the antibacterial activities of CDs via bacterial growth inhibition assays. As shown in Fig. 1a, b, the growth of *E. coli* and *S. aureus* was determined by the dose of CDs, the bacterial viability gradually decreased with concentrations increasing from 8 to 16, 32, 64, 128, and 256 μg/mL. CD110 could partly inhibit the proliferation of *E. coli* and and *S. aureus* until the concentration was up to 256 and 64 μg/mL, respectively. By contrast, CD1100 exhibited no bacteriostatic effect within the experimental concentration range (Additional file 1: Fig. S2 and S3). Among the CDs, CD31 exhibited the strongest bactericidal activity and its antibacterial activity was further estimated by colony-forming unit (CFU) counting assay. As depicted in Fig. 1c, the number of colonies of the two strains apparently decreased with the concentrations of CD31 rising from 8 to 256 μg/mL. The minimum inhibitory concentration (MIC) of CD31 against *E. coli* and *S. aureus* was 64 and 32 μg/mL, respectively, demonsreating CD31 is a broad-spectrum and efficient sterilant. The antibacterial efficacy of Plys was assessed in a similar way, the survival rate of *E. coli* and *S. aureus* lowered with increasing the dose of Plys (Additional file 1: Fig. S4), and the MIC of Plys toward the two bacterial strains was 128 µg/mL (Additional file 1: Fig. S5). The cytocompatibility of CD31 (Additional file 1: Fig. S6) and Plys (Additional file 1: Fig. S7) was evaluated toward L929 cells by MTT assay. All of the cell viabilities were over 89%, even the concentration was as high as 400 µg/mL, verifying both CD31 and Plys have favorable biocompatibility. Robust bactericidal activities as well as excellent biocompatibility make CD31 and Plys great candidates for antibacteria.

### Characterization of CD31

**Fig. 2:**
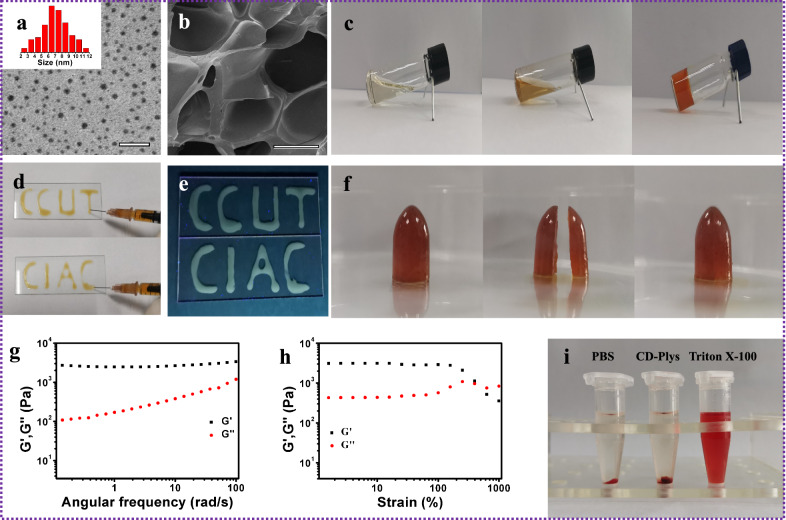
**a** TEM analysis of CD31. The insert is the size distribution of CD31. **b** SEM image of CD-Plys hydrogel. Scale bar: 200 nm. **c** Photographs of the Plys solution (400 mg/mL), the CD31 solution (200 mg/mL), and CD-Plys hydrogel (Plys = 400 mg/mL, CD31 = 200 mg/mL) at room temperature. **d** The injectability of CD-Plys hydrogel under **d** natural light and **e** UV (365 nm) light. **f** Self-healing performance of CD-Plys hydrogel. **g** Frequency sweep measurement for CD-Plys hydrogel. **h** Strain sweep measurement for CD-Plys hydrogel at fixed angular frequency of 1 Hz. **i** Hemolytic activity of CD-Plys hydrogel

Transmission electron microscopy (TEM) image (Fig. 2a) displays that CD31 is spherical with the average diameter of 6.96 ± 0.3 nm. CD31 has broad absorption in the UV–vis region with two typical absorption band at 233 nm and 292 nm (Additional file 1: Additional file 1: Fig. S8), the former is ascribed to π-π* transition (C = C) and the latter corresponds to n-π* transition (C = O). The emission spectra of CD31 (Additional file 1: Fig. S9a) indicate that CD31 has the maximum emission at 483 nm under the optimal excitation of 400 nm. Fourier transform infrared (FT-IR) spectrum (Additional file 1: Fig. S10) of CD31 exhibit peaks at 3000–2800 cm^−1^ (–OH and –CH_2_), ~ 1700 cm^−1^ (–CHO) and ~ 1100 cm^−1^ (–C–O). The X-ray diffraction pattern (XRD) of CD31 is depicted in Additional file 1: Fig. S11, and the peak centered at 24.2° confirms the crystalline feature of CD31.

### Synthesis and characterization of CD-Plys hydrogel

The gelation process of CD-Plys was shown in Fig. 1c, the aqueous solution of Plys (400 mg/mL) was mixed with CD31 aqueous solution at a mass ratio of 2:1. The mixture of Plys and CD31 stood at room temperature for 40 min. In the process, the aldehyde groups on CD31 covalently linked the amino groups of Plys via Schiff-base reaction to form CD-Plys hydrogel, and the hydrogel viscosity was verified by the method from “stable to inversion” (Fig. 2c). The emission spectra of CD-Plys hydrogel are shown in Additional file 1: Fig. S9b, the maximum emission of CD-Plys is observed at 530 nm, which is red-shifted by 45 nm with respect to that of CDs. FT-IR spectra were conducted to elucidate the formation of CD-Plys hydrogel (Additional file 1: Fig. S10). The FT-IR spectrum of CD-Plys hydrogel has the characteristic vibration of imine bonds (1658 cm^−1^), along with the weakening of characteristic peak (1700 cm^−1^) of aldehyde groups, indicating the successful conjugation of the aldehyde groups in CD31 with the amino groups in Plys. The XRD spectrum of Plys presents a strong spike at 27.4° and a broad band at 40.3°, while CD-Plys has two broad characteristic peaks at 25.8° and 39.5° (Additional file 1: Fig. S11), verifying that CD-Plys was resoundingly synthesized from CD31 and Plys. The zeta potential of CD31, Plys and CD-Plys is -9.0, 9.29 and 9.18 mV, respectively, further proving the successful preparation of CD-Plys hydrogel (Additional file 1: Fig. S12).

The scanning electron microscopy (SEM) analysis reveals that CD-Plys hydrogel possesses porous structure, which can be attributed to the interwoven network between CD31 and Plys (Fig. 2b). The inherent porous structure of CD-Plys hydrogel can significantly enhance the area of contact with wound, and quickly absorb blood and tissue exudates. In addition, extrusion experiment was carried out to assess the injectability of CD-Plys gel. As shown in Fig. 2d and1e, CD-Plys hydrogel could be continuously injected on the glass slide through the syringe needle, wrote the letter “CCUT” (referring to Changchun University of Technology) and “CIAC” (referring to Changchun Institute of Applied Chemistry). The photographs in Fig. 2f demonstrate the self-repairing properties of CD-Plys hydrogel. The cutted two pieces could fuse together within 30 min after they touched with each other. Since imine bonds in the network are reversible, CD-Plys could be cut in half readily. Once these two fragments are in contact with each other, the amino groups on the fracture surface will react quickly with the contacted aldehyde groups and regenerate imine bonds, to reconfigure the hydrogel matrix for self-repairing, thereby inducing the robust self-healing capability of CD-Plys. Rheological analysis under different frequencies and strains was carried out in order to verify the mechanical property of CD-Plys. As depicted in Fig. 2g, the storage modulus (G') values are much higher than the loss modulus (G'') in the the whole frequency sweep range, confirming that CD-Plys does possess a well-developed 3D network. As the frequency increases, the maximum G' value reaches 3.3 kpa, indicating that CD-Plys has relatively high mechanical strength. Furthermore, we investigated the thixotropic behavior of CD-Plys by a dynamic rheometer. Figure 2h shows that the hydrogel structure of CD-Plys is not broken until the strain exceeds 400%. The hemolytic toxicity of CD-Plys was evaluated toward red blood cells (RBCs) and shown in Fig. 2i and Additional file 1: Fig. S13. Only 1.4% of RBCs lysed in CD-Plys hydrogel group, indicating that CD-Plys has good hemocompatibility. The cytocompatibility of CD-Plys toward L929 cells was assessed by MTT assay (Additional file 1: Fig. S14). The cell viability was over 95% even after direct contact with CD-Plys for 48 h. The excellent biological safety endows CD-Plys hydrogel with great potential in biomedical applications.

### In vitro antibacterial of CD-Plys hydrogel

**Fig. 3:**
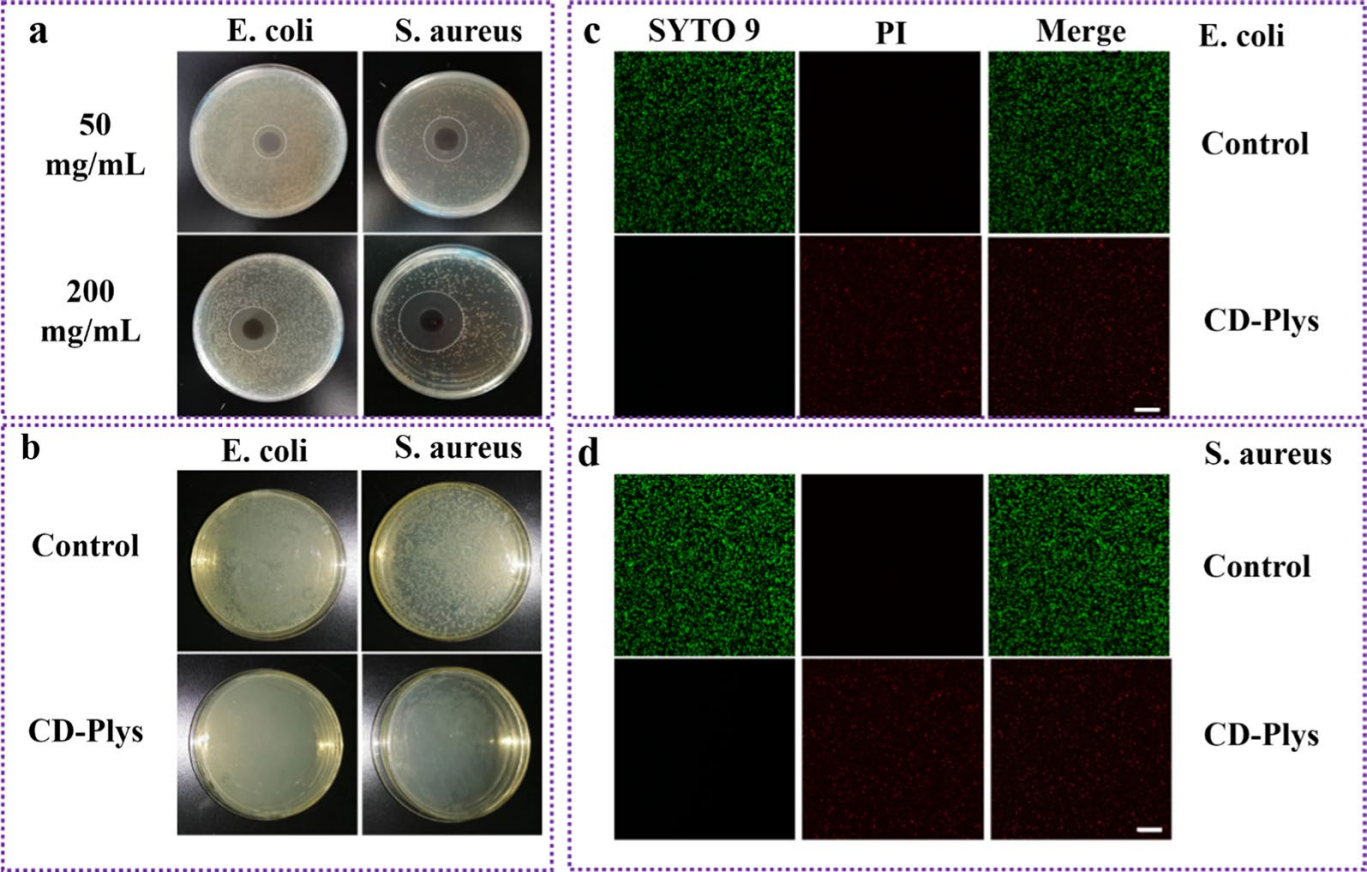
In vitro antibacterial assay of CD-Plys hydrogel. **a** Antibacterial sensitivity of CD-Plys hydrogel against *E. coli* and *S. aureus* with agar diffusion tests at 24 h. **b** LB agar inoculated with uncoated or CD-Plys hydrogel coated substrates. The white dots are colonies of live bacteria in the plates. SYTO 9/PI staining images of *E. coli* **c** and *S. aureus* **d** treated without/with CD-Plys hydrogel. Scale bar, 20 µm

The antibacterial activity of CD-Plys hydrogel against *E. coli* and *S. aureus* was assessed by the standard agar disc diffusion assay. When *E. coli* or *S. aureus* was treated with CD-Plys hydrogel that prepared from various concentrations (50 or 200 mg/mL) of CDs and Plys (200 mg/mL) at 37 °C for 24 h, distinct inhibition zones were formed (Fig. 3a). For 200 mg/mL of CDs, the zone diameter in *E. coli* and *S. aureus* group is about 2.0 ± 0.05 cm and 3.0 ± 0.06 cm, respectively. For 50 mg/mL of CDs, the zone diameter in *E. coli* group and *S. aureus* group is about 1.0 ± 0.02 cm and 1.7 ± 0.02 cm, respectively. The results demonstrated CD-Plys had an excellent broad-spectrum antibacterial efficacy against both *E. coli* and *S. aureus* and its antibacterial activity was proportional to the concent of CDs. Moreover, CD-Plys exhibits higher antibacterial activity on *S. aureus* than *E. coli*. Then the contacting-antibacterial ability of CD-Plys was assessed against *E. coli* and *S. aureus*, by coating CD-Plys on the surface of agar plates. The bacteria cultivation without CD-Plys was set as the control. As depicted in Fig. 3b, almost no *E. coli* or *S. aureus* colonies were proliferated for the bacterial suspension collected from the substrate coated with CD-Plys, while bacteria colonies were observed obviously for the control group. These results confirm that CD-Plys can be used as a local antibacterial coating with antibacterial effect. In order to further assess the antibacterial activity of CD-Plys, we performed live/dead staining assays by using SYTO 9 green dye and propidium iodide (PI) red dye to stain live and dead bacteria, respectively. *E. coli* and *S. aureus* were incubated with CD-Plys for 12 h, and then costained by SYTO 9 and PI. As depicted in Fig. 3c, d, the bacteria in the control groups emitted intense green fluorescence, indicating that they were alive. By contrast, the bacteria in the CD-Plys groups displayed strong red signals, implying that all of them were eradicated by CD-Plys.

### Antibacterial mechanism of CD31 and CD-Plys

**Fig. 4:**
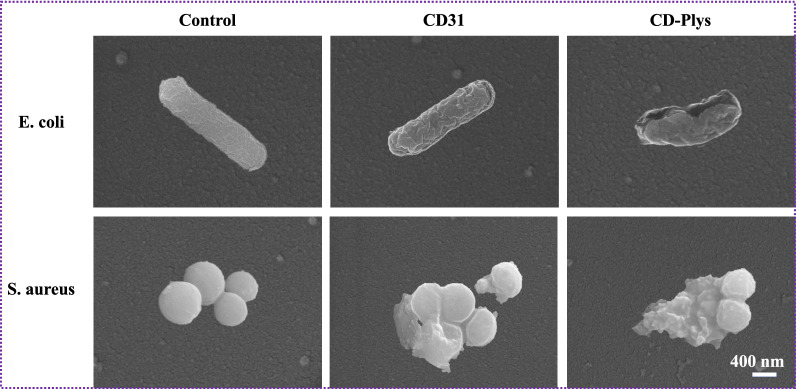
SEM images of *E. coli* and *S. aureus* treated with CD31 and CD-Plys. Bacteria without treatment were set as controls

SEM was utilized to study the bacteria’s morphological changes after treatment with CD31 and CD-Plys (Fig. 4). Without treatment, the bacteria had regular shapes with intact membrane and clear edges. After incubation with CD31, the membranes of *E. coli* shrank and cracked, while *S. aureus* were disrupted and the cytoplasm outflowed. As we all know, the surface properties of nanoantibiotics directly affect their interaction with bacteria. Although the zeta potential of CD31 was negative, the aldehyde groups could irreversibly destroy the bacterial cell wall and the cytoplasmic membrane, eventually leading to the bacterial death. As for the bacteria treated with CD-Plys, more serious phenomena of shrinkage, collapse, cracking, fusing together and leakage of intracellular components were observed, verifying that CD-Plys could seriously damaged the bacterial cells.

### In vivo antibacterial and skin wound healing of CD-Plys hydrogel

**Fig. 5:**
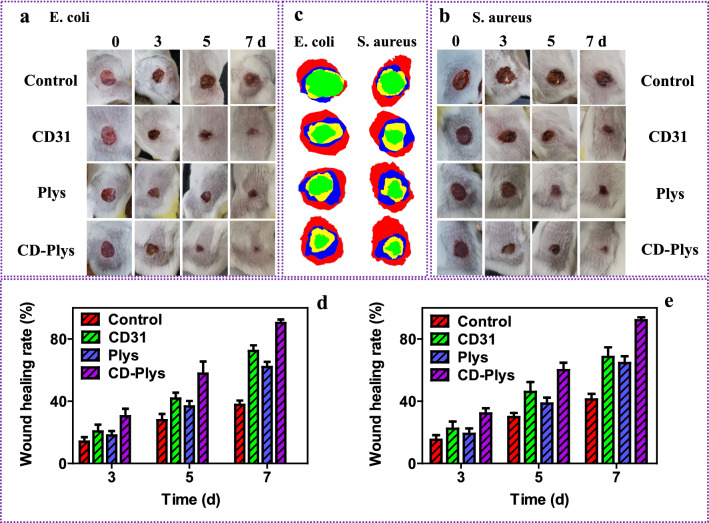
In vivo infected wound healing evaluation. **a** Time-dependent images of *E. coli* infected wounds. **b** Time-dependent images of *S. aureus* infected wounds. **c** Traces of wound area during 7 days of treatments (0 days: red pattern; 3 days: blue pattern; 5 days: yellow pattern; 7 days: green pattern). Wound healing rate of **d** *E. coli* or **e** *S. aureus* infected wound at different time points, data are presented as mean with standard deviation as error bars (*n* = 6)

The favorable antibacterial capacity and good cytocompatibility of CD-Plys encouraged us to evaluate the bacteria inhibition and wound healing efficiency of CD-Plys in vivo. Full-thickness wound defect models with round skin injuries (8 mm in diameter) were created on the back of mice. Afterward, 48 mice were randomly divided into 8 groups. Mice in the four groups were infected by *E. coli*, while the others were infected by *S. aureus*. The wound healing processes under different conditions were monitored (Fig. 5a, b), and traces of wound closure were drawn during 7 days of treatment (Fig. 5c). The wound healing rates at 3, 5, and 7 days were evaluated and depicted in Fig. 5d, e. For all of these mice, the wound size decreased gradually with time. On the third day, the epidermis of infected wounds in the two CD-Plys groups began to regenerate. On the seventh day, the two groups mice treated with CD-Plys exhibited the best wound healing, the wound were nearly closed with the healing rate of 90% (*E. coli*) and 92% (*S. aureus*), respectively, moreover, a lot of hair grew out. The results confirmed that the enhanced antibacterial activity of CD-Plys was the synergistic effect of CD31 and Plys, which could significantly accelerate skin wound closure and tissue regeneration.

**Fig. 6:**
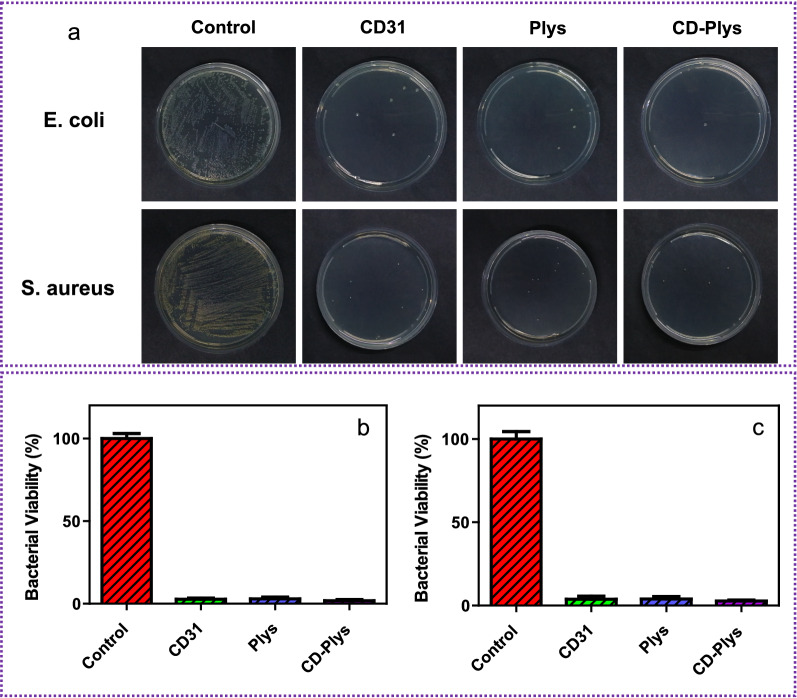
In vivo assessment of CD-Plys hydrogel with antibacterial effects on infected wounds and skin wound healing capability. **a** The *E. coli* and *S. aureus* bacteria were obtained from wound tissues and incubated on LB agar plates. The percentage of **b** *E. coli* and **c** *S. aureus* colonies appeared on the LB agar plate to the control groups (*n *= 6)

After 24 h of treatment, the residual bacteria were extracted from the wound tissue, incubated with LB medium for 24 h, and then measured by standard plate counting methods (Fig. 6a). There were nearly no bacterial colonies in the wound tissues those were treated with CDs, Plys or CD-Plys hydrogel. The statistical analysis of Fig. 6a was shown in Fig. 6b (*E. coli*) and Fig. 6c (*S. aureus*), respectively. The results suggest that CD-Plys hydrogel exhibits the strongest antibacterial capability.

**Fig. 7:**
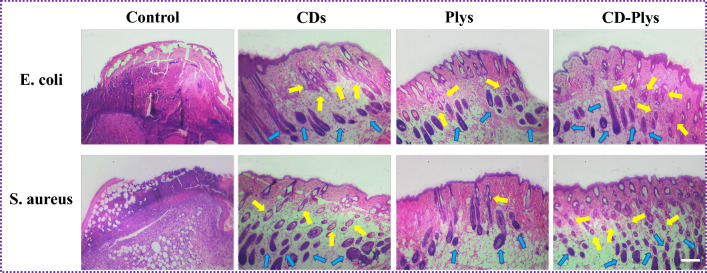
H&E stained images of skin tissues treated with PBS, CD31, Plys and CD-Plys, respectively. (blood vessels: yellow arrows; hair follicles: blue arrows). Scale bar: 200 µm

Histological analysis was used to evaluate the quality of regenerated wound tissue. After 7 days of treatment, the tissues were dissected and stained with hematoxylin and eosin (H&E) (Fig. 7). *E. coli* and *S. aureus* infected tissues in the control groups showed severe inflammatory cell infiltration. For CD31 and Plys groups, a few new blood vessels and hair follicles were observed. The CD-Plys groups exhibit the well-organized lamellar epithelium and orderly granulation tissue, with a large number of new blood vessels and hair follicles. The results of tissue sections indicated that CD-Plys could be used as an effective wound dressing material for treatment of bacterial infections and facilitating skin wound healing process.

## Conclusions

All in all, negatively charged CD31 with potent antimicrobial activity was synthesized from GA and PEG_200_, which could effectively destroy *E. coli* and *S. aureus* with MIC of 64 and 32 μg/mL, respectively. Then CD31 reacted with Plys to fabricate CD-Plys hydrogel. CD-Plys could integrate the favorable attributes from CD31 and Plys, and achieve synergy antibacterial effect of “one plus one greater than two”. Combined with the fascinating features of favorable injectability, eximious self-healing, excellent biocompatibility and exceptional broad-spectrum antimicrobial activity, CD-Plys could significantly promote full-thickness cutaneous wound healing with accelerated wound closure and improved skin regeneration. Overall, this study provides a new strategy for fabricating outstanding antibacterial wound dressing to efficiently promote the healing of microbial infection wounds.

## Supplementary Information

**Additional file 1: Figure S1.** Zeta potential of CDs. **Figure S2.** Antimicrobial assay of CD110 and CD1100 against *E. coli*. **Figure S3.** Antimicrobial assay of CD110 and CD1100 against *S. aureus*. **Figure S4.** Antimicrobial assay of Plys toward *E. coli* and *S. aureus*. **Figure S5.** Antimicrobial assay of Plys toward *S. aureus* with LB agar. **Figure S6.** MTT assay of CD31 toward L929 cells. **Figure S7.** MTT assay of Plys toward L929 cells. **Figure S8.** UV-vis spectrum of CD31 in deionized water. **Figure S9.** Photoluminescent spetra of (a) CD31 aqueous solution and (b) CD-Plys under the excitation of different wavelengths. **Figure S10.** FT-IR spectra of CD31 (black solid line), Plys (red solid line) and CD-Plys (blue solid line). **Figure S11.** The X-ray diffraction pattern of CD31 (black), Plys (red) and CD-Plys hydrogel (blue). **Figure S12.** Zeta potentials of CD31, Plys and CD-Plys hydrogel. **Figure S13.** The hemolysis assay of PBS, CD-Plys and Triton X-100. **Figure S14.** MTT assay of CD-Plys with direct contact with L929 cells for 12, 24 and 48 h.

## Data Availability

Availability of data and materials is available from the journal or from the author.
