# Supplementary material for: Injectable self-healing hydrogel fabricated from antibacterial carbon dots and ɛ-polylysine for promoting bacteria-infected wound healing
Source: J Nanobiotechnology. 2022 Aug 11;20:368. doi: 10.1186/s12951-022-01572-w (PMC9367091; doi:10.1186/s12951-022-01572-w)
Supplement: Supplementary file 1 — Additional file 1: Figure S1. Zeta potential of CDs. Figure S2. Antimicrobial assay of CD110 and CD1100 against E. coli. Figure S3. Antimicrobial assay of CD110 and CD1100 against S. aureus. Figure S4. Antimicrobial assay of Plys toward E. coli and S. aureus. Figure S5. Antimicrobial assay of Plys toward S. aureus with LB agar. Figure S6. MTT assay of CD31 toward L929 cells. Figure S7. MTT assay of Plys toward L929 cells. Figure S8. UV-vis spectrum of CD31 in deionized water. Figure S9. Photoluminescent spetra of (a) CD31 aqueous solution and (b) CD-Plys under the excitation of different wavelengths. Figure S10. FT-IR spectra of CD31 (black solid line), Plys (red solid line) and CD-Plys (blue solid line). Figure S11. The X-ray diffraction pattern of CD31 (black), Plys (red) and CD-Plys hydrogel (blue). Figure S12. Zeta potentials of CD31, Plys and CD-Plys hydrogel. Figure S13. The hemolysis assay of PBS, CD-Plys and Triton X-100. Figure S14. MTT assay of CD-Plys with direct contact with L929 cells for 12, 24 and 48 h. [file 12951_2022_1572_MOESM1_ESM.docx]

Additional file Information

Injectable self-healing hydrogel fabricated from antibacterial carbon dots and ɛ-polylysine for promoting bacteria-infected wound healing

Chengjian Mou, ^a^ Xinyuan Wang, ^a^ Jiahui Teng, ^a^ Zhigang Xie, ^b,^* Min Zheng, ^a,^*

*^a^School of Chemistry and Life Science, Advanced Institute of Materials Science, Changchun University of Technology, 2055 Yanan Street, Changchun, Jilin 130012, P. R. China*

*^b^State Key Laboratory of Polymer Physics and Chemistry, Changchun Institute of Applied Chemistry, Chinese Academy of Sciences, 5625 Renmin Street, Changchun, Jilin 130022, P. R. China.*

**Contents:**

**Figure S1.** Zeta potential of CDs.

**Figure S2.** Antimicrobial assay of CD110 and CD1100 against *E. coli*.

**Figure S3.** Antimicrobial assay of CD110 and CD1100 against *S. aureus*.

**Figure S4.** Antimicrobial assay of Plys toward *E. coli* and *S. aureus*.

**Figure S5.** Antimicrobial assay of Plys toward *S. aureus* with LB agar.

**Figure S6.** MTT assay of CD31 toward L929 cells.

**Figure S7.** MTT assay of Plys toward L929 cells.

**Figure S8.** UV-vis spectrum of CD31 in deionized water.

**Figure S9.** Photoluminescent spetra of (a) CD31 aqueous solution and (b) CD-Plys under the excitation of different wavelengths.

**Figure S10.** FT-IR spectra of CD31 (black solid line), Plys (red solid line) and CD-Plys (blue solid line).

**Figure S11.** The X-ray diffraction pattern of CD31 (black), Plys (red) and CD-Plys hydrogel (blue).

**Figure S12.** Zeta potentials of CD31, Plys and CD-Plys hydrogel.

**Figure S13.** The hemolysis assay of PBS, CD-Plys and Triton X-100.

**Figure S14.** MTT assay of CD-Plys with direct contact with L929 cells for 12, 24 and 48 h.

**Figure S1.** Zeta potential of CDs.


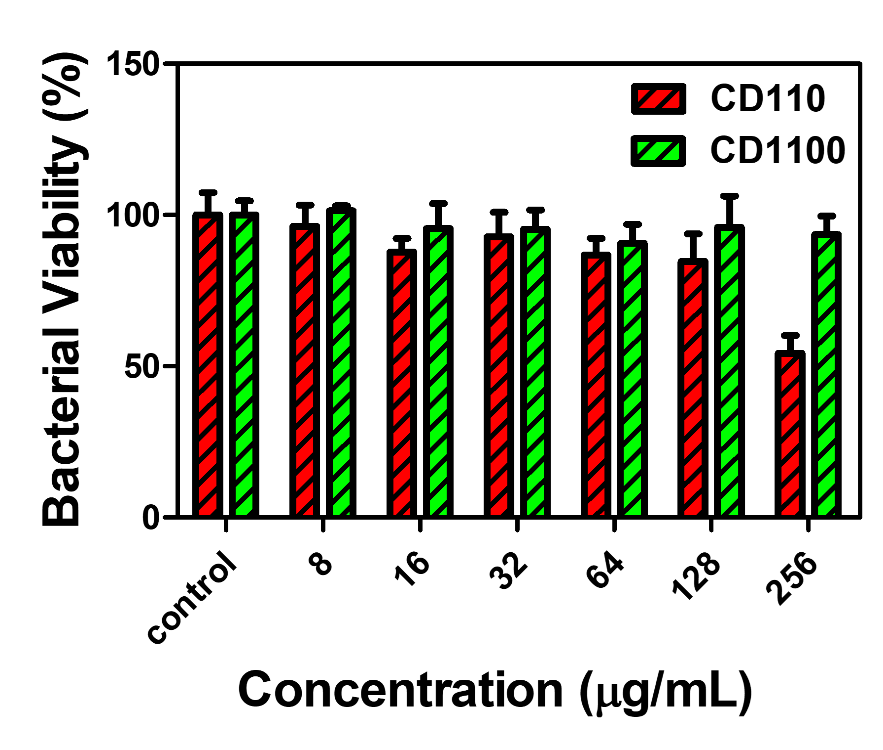


**Figure S2.** Antimicrobial assay of CD110 and CD1100 against *E. coli*.


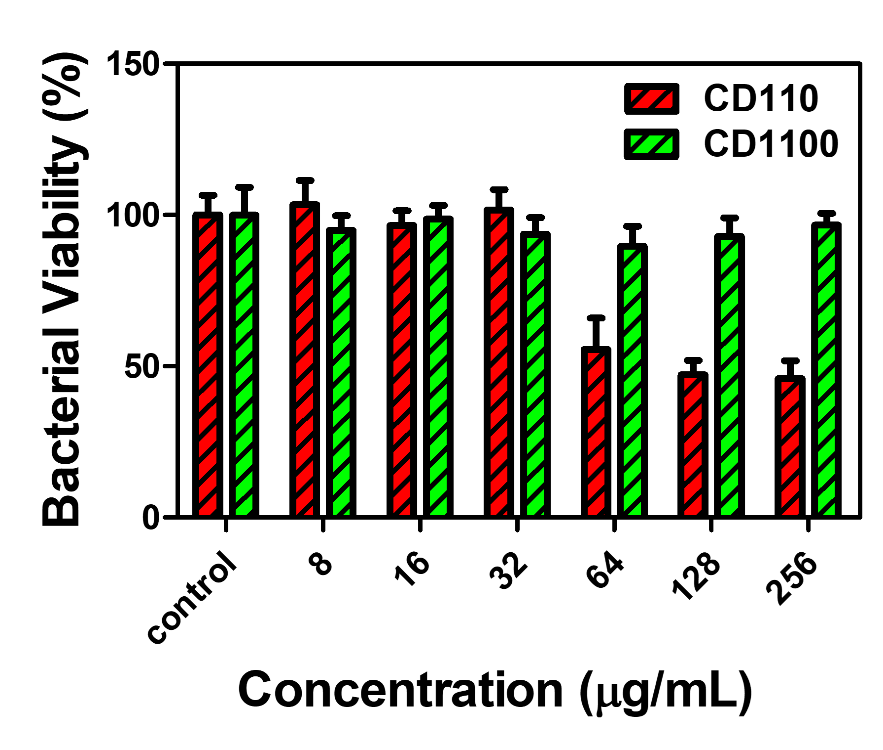


**Figure S3.** Antimicrobial assay of CD110 and CD1100 against *S. aureus*.


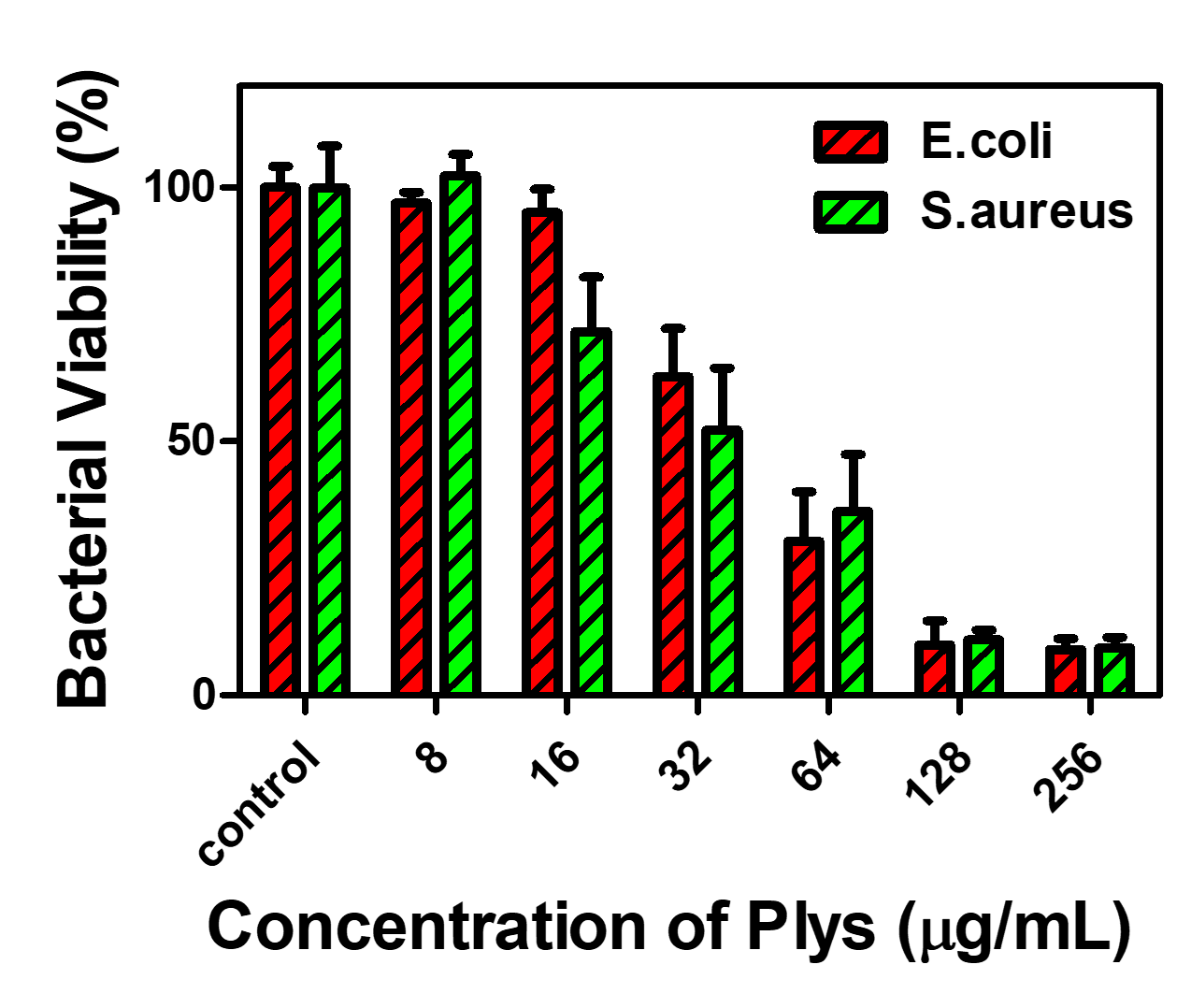


**Figure S4.** Antimicrobial assay of Plys toward *E. coli* and *S. aureus*.

**
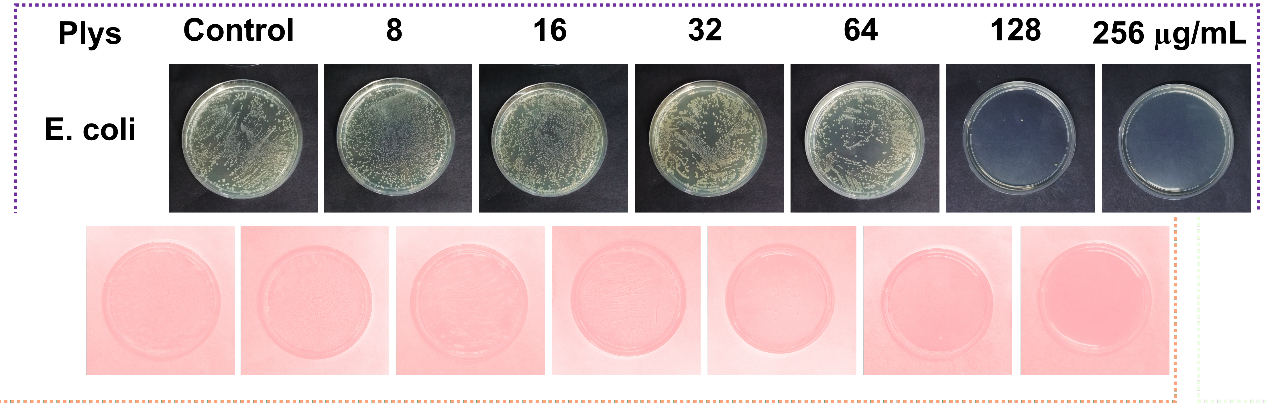
**

**Figure S5.** Antimicrobial assay of Plys toward *S. aureus* with LB agar.

**Figure S6.** MTT assay of CD31 toward L929 cells.


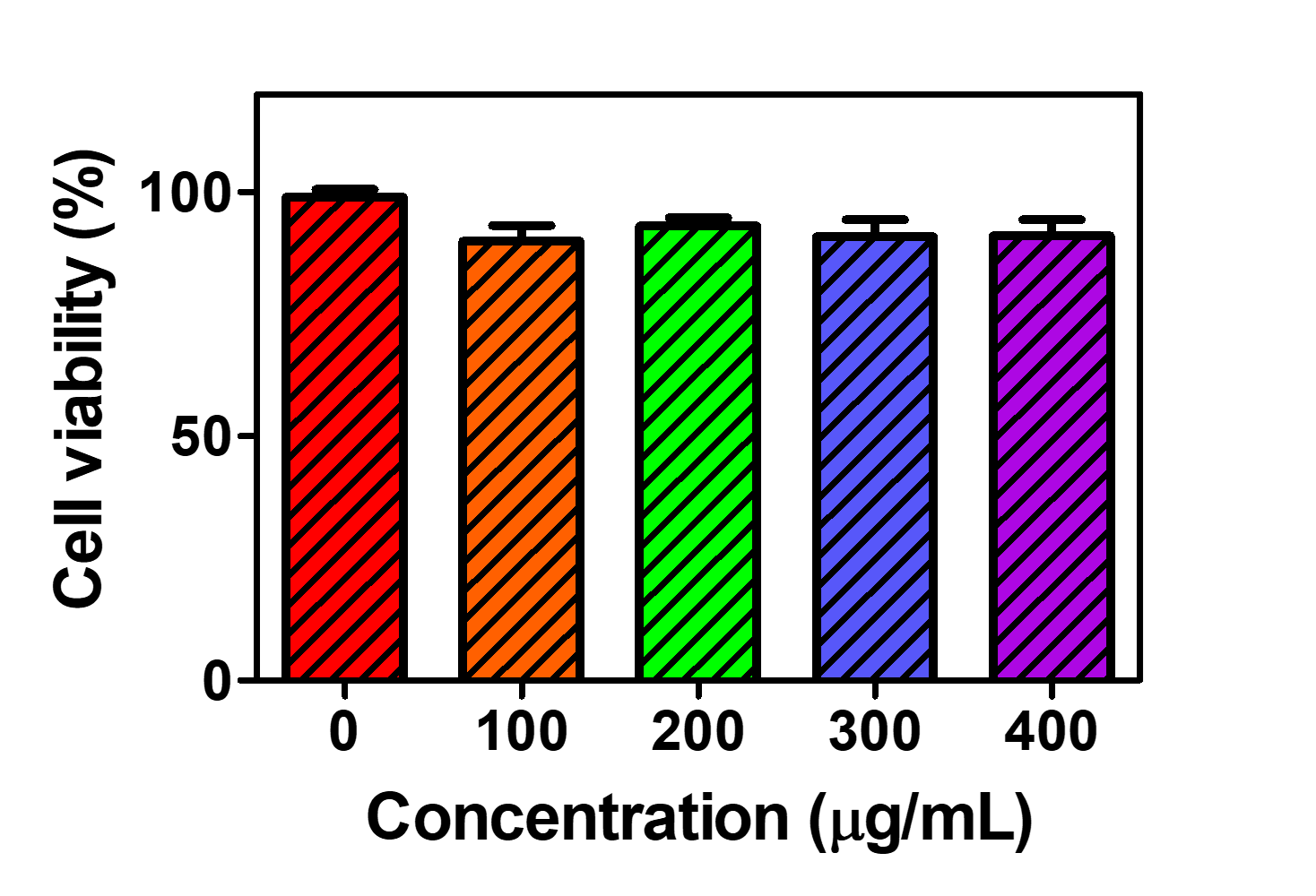


**Figure S7.** MTT assay of Plys toward L929 cells.


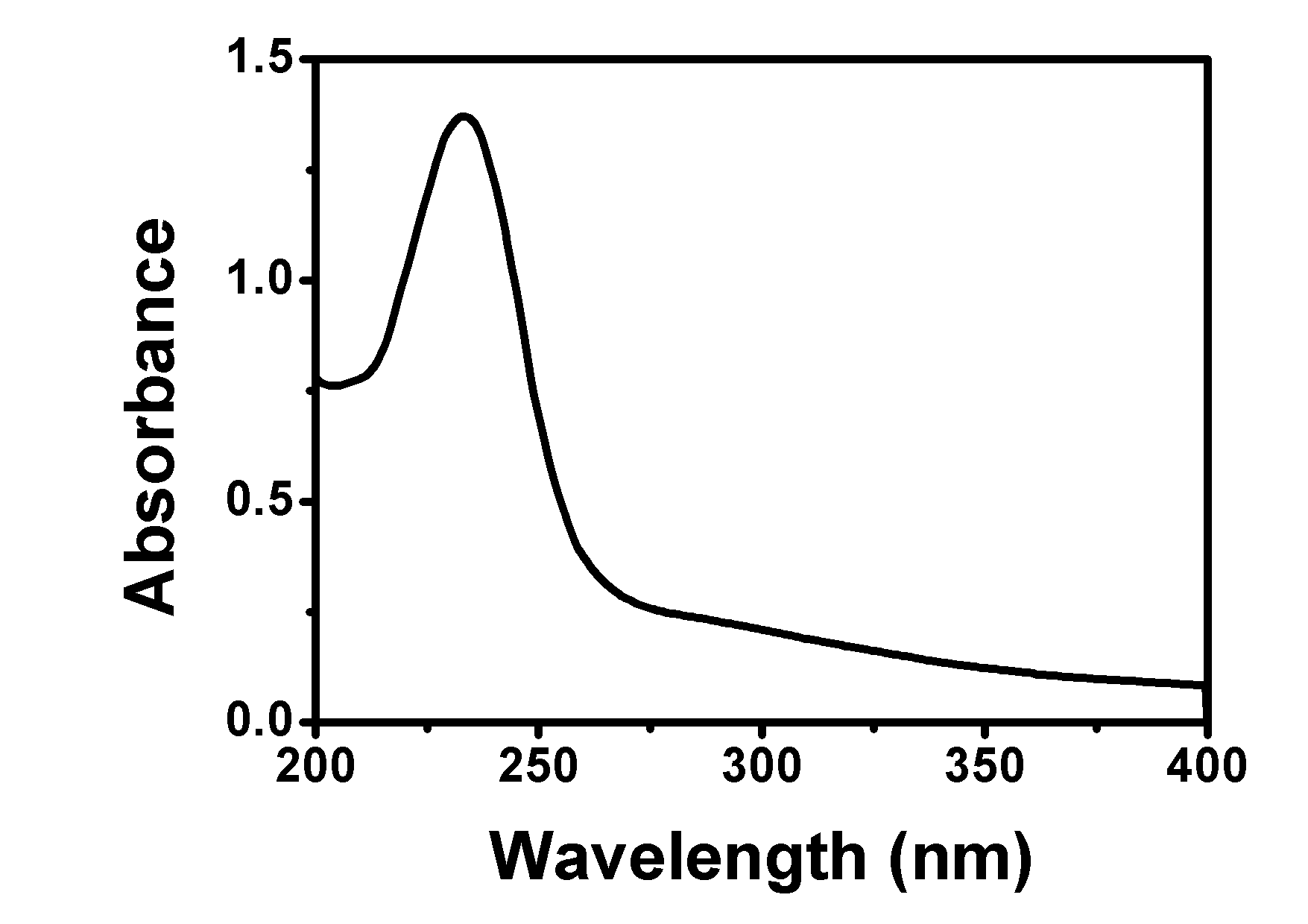


**Figure S8.** UV-vis spectrum of CD31 in deionized water.


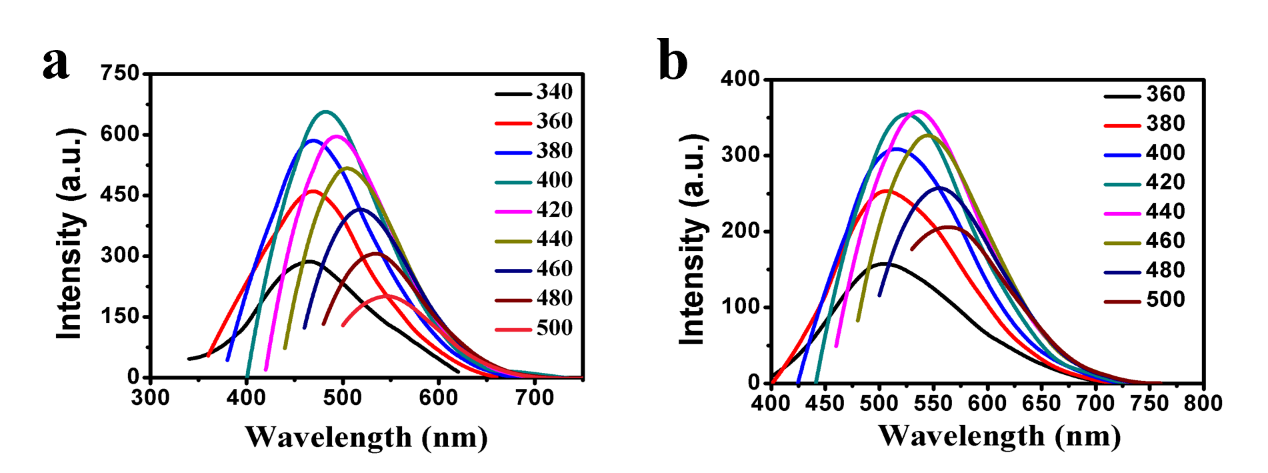


**Figure S9.** Photoluminescent spetra of (a) CD31 aqueous solution and (b) CD-Plys under the excitation of different wavelengths.

**
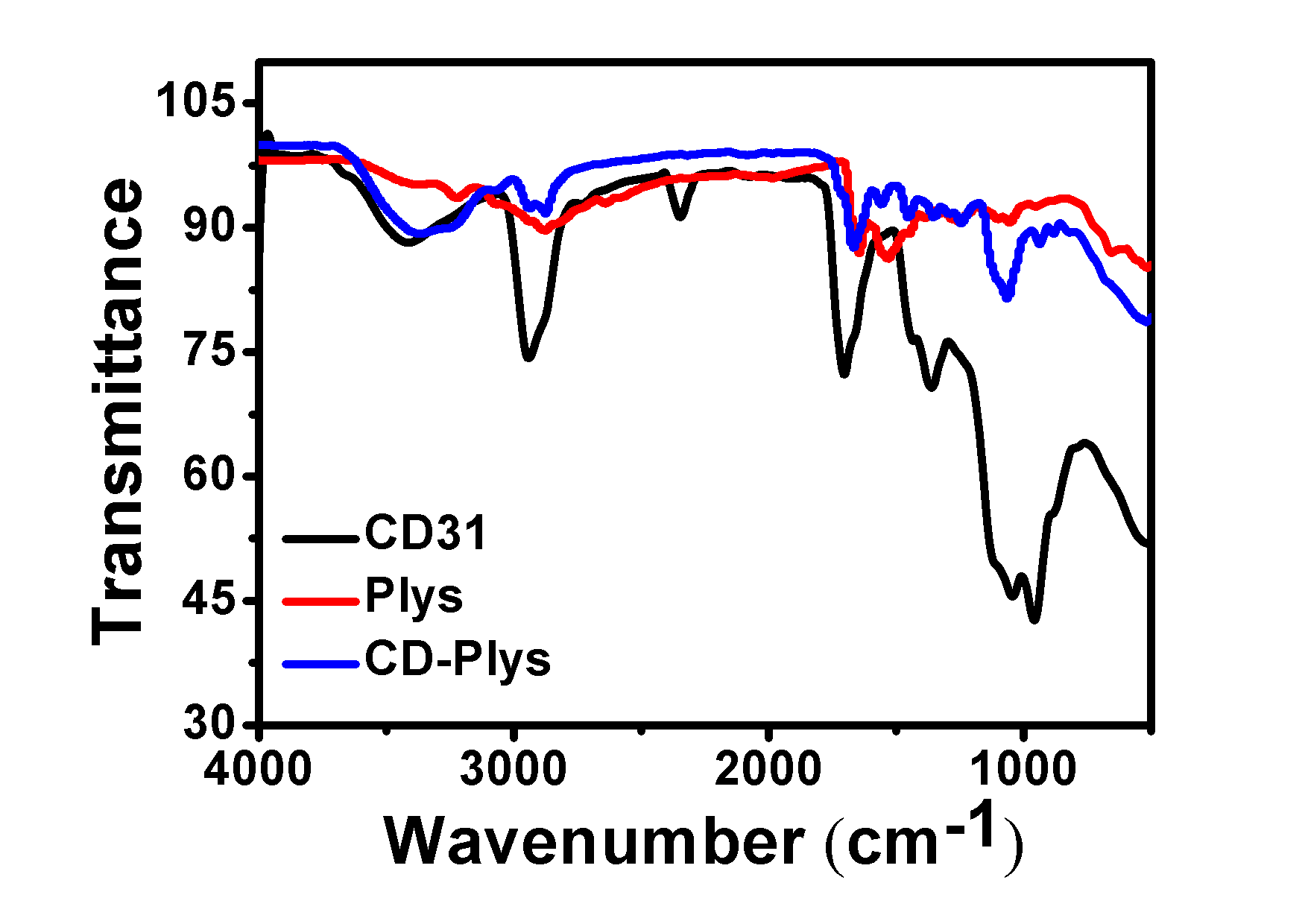
**

**Figure S10.** FT-IR spectra of CD31 (black solid line), Plys (red solid line) and CD-Plys (blue solid line).

**
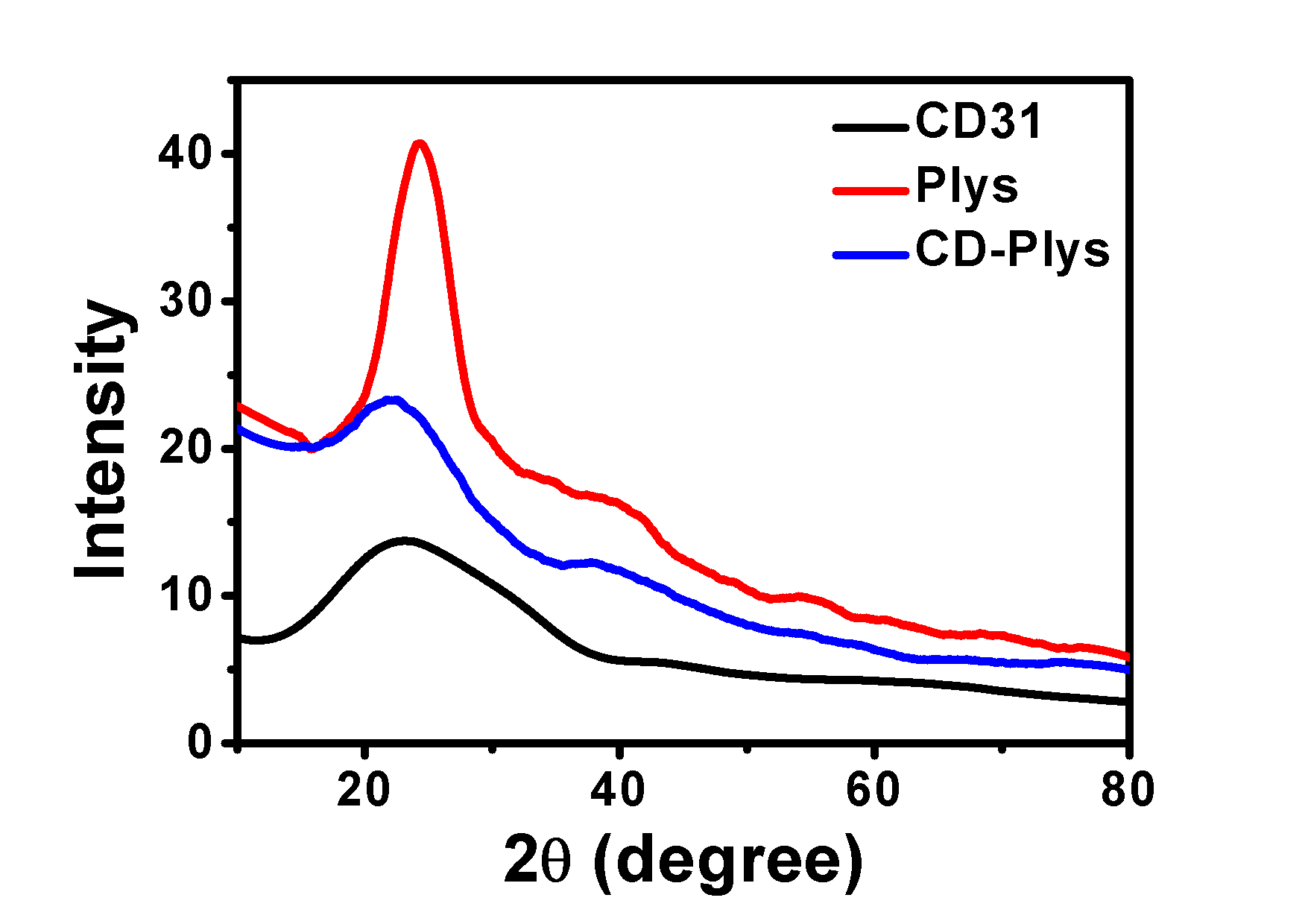
**

**Figure S11.** The X-ray diffraction pattern of CD31 (black), Plys (red) and CD-Plys hydrogel (blue).


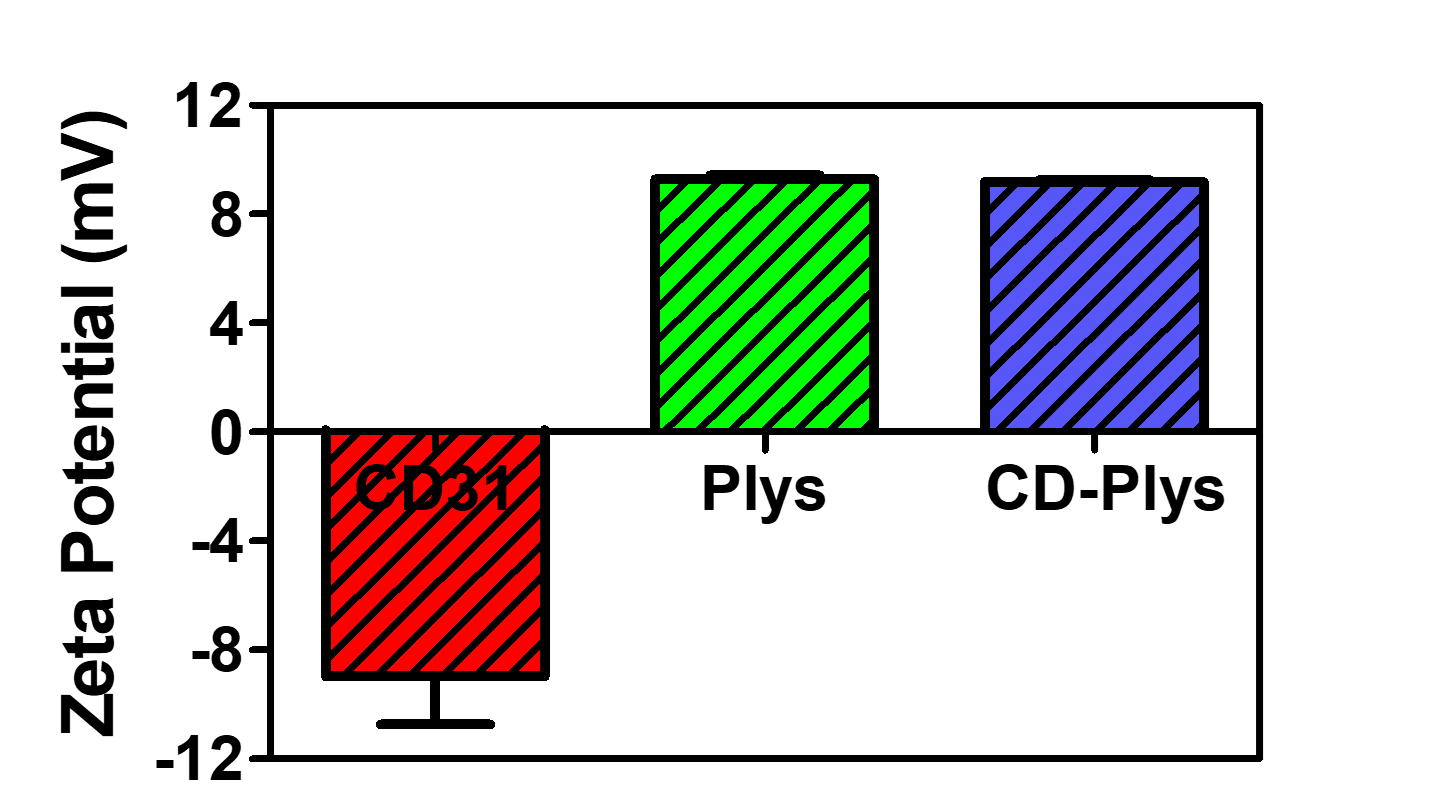


**Figure S12.** Zeta potentials of CD31, Plys and CD-Plys hydrogel.


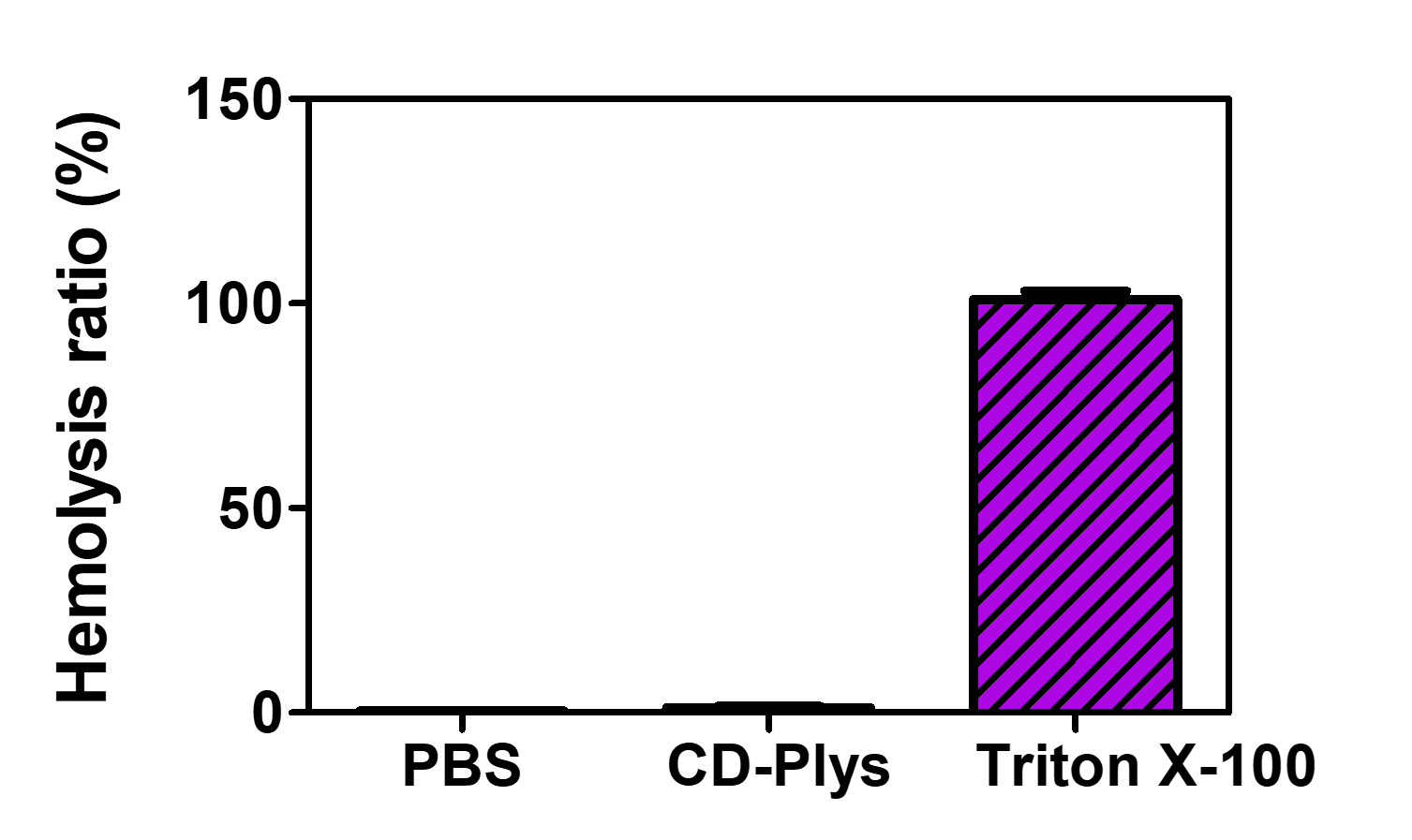


**Figure S13.** The hemolysis assay of PBS, CD-Plys and Triton X-100.


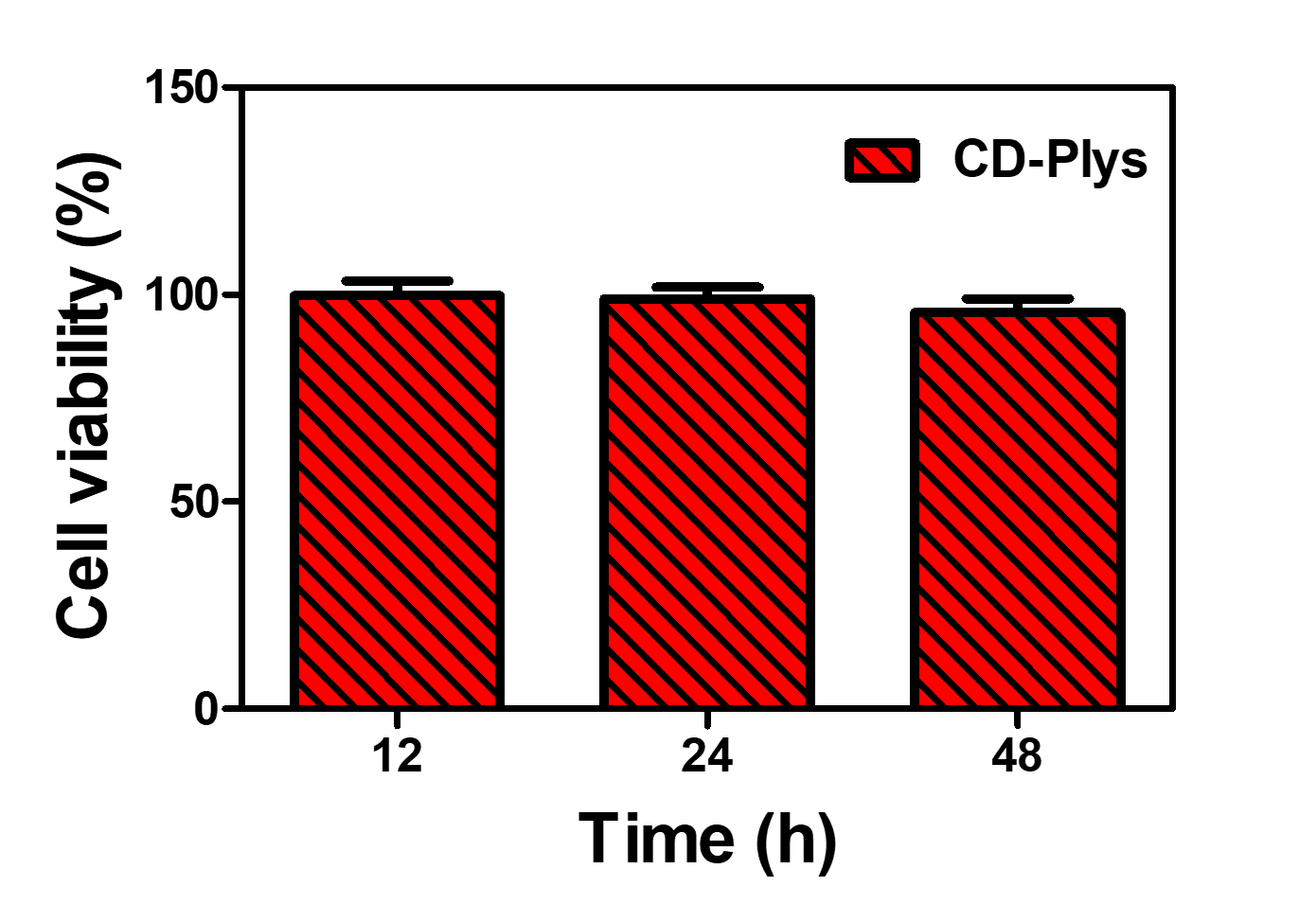


**Figure S14.** MTT assay of CD-Plys with direct contact with L929 cells for 12, 24 and 48 h.
